# Analyses of competent and non‐competent subpopulations of *Bacillus subtilis* reveal *yhfW*, *yhxC* and ncRNAs as novel players in competence

**DOI:** 10.1111/1462-2920.15005

**Published:** 2020-04-15

**Authors:** Mirjam Boonstra, Marc Schaffer, Joana Sousa, Luiza Morawska, Siger Holsappel, Petra Hildebrandt, Praveen Kumar Sappa, Hermann Rath, Anne de Jong, Michael Lalk, Ulrike Mäder, Uwe Völker, Oscar P. Kuipers

**Affiliations:** ^1^ Molecular Genetics group, Groningen Biomolecular Sciences and Biotechnology Institute (GBB) University of Groningen the Netherlands; ^2^ Department of Functional Genomics, Interfaculty Institute of Genetics and Functional Genomics University Medicine Greifswald Germany; ^3^ Department of Cellular Biochemistry/Metabolomics, Institute of Biochemistry University of Greifswald Germany

## Abstract

Upon competence‐inducing nutrient‐limited conditions, only part of the *Bacillus subtilis* population becomes competent. Here, we separated the two subpopulations by fluorescence‐assisted cell sorting (FACS). Using RNA‐seq, we confirmed the previously described ComK regulon. We also found for the first time significantly downregulated genes in the competent subpopulation. The downregulated genes are not under direct control by ComK but have higher levels of corresponding antisense RNAs in the competent subpopulation. During competence, cell division and replication are halted. By investigating the proteome during competence, we found higher levels of the regulators of cell division, MinD and Noc. The exonucleases SbcC and SbcD were also primarily regulated at the post‐transcriptional level. In the competent subpopulation, *yhfW* was newly identified as being highly upregulated. Its absence reduces the expression of *comG*, and has a modest, but statistically significant effect on the expression of *comK*. Although expression of *yhfW* is higher in the competent subpopulation, no ComK‐binding site is present in its promoter region. Mutants of *yhfW* have a small but significant defect in transformation. Metabolomic analyses revealed significant reductions in tricarboxylic acid (TCA) cycle metabolites and several amino acids in a Δ*yhfW* mutant. RNA‐seq analysis of Δ*yhfW* revealed higher expression of the NAD synthesis genes *nadA*, *nadB* and *nadC*.

## Introduction

*Bacillus subtilis* is a Gram‐positive soil bacterium capable of developing natural competence. During competence, cell division and replication are halted and the cell can take up exogenous DNA from the environment (Haijema *et al*., 2001; Briley *et al*., 2011; Mirouze *et al*., 2015) Under nutrient‐limited conditions in the lab, approximately 5%–50% of a *B. subtilis* 168 population becomes competent. The main regulator of competence is ComK, which binds to K‐boxes within the promoter region of competence genes, thereby altering the expression of the downstream genes (van Sinderen *et al*., 1994, 1995; Hamoen *et al*., 1998). The competence state (K‐state) of *B. subtilis* has previously been studied with microarray techniques and LacZ fusions (Hamoen *et al*., 1998; Berka *et al*., 2002; Ogura *et al*., 2002) To overcome the problem posed by the smaller fraction of competent cells, these studies compared *comK* and/or *mecA* deletion mutants with wild‐type (WT) strains. Deletion of *comK* prevents competence, whereas deletion of *mecA* prevents degradation of *comK* and inhibits exit from competence (Hahn *et al*., 1995; Turgay *et al*., 1998). In the transcriptomics studies, no significant downregulation of genes by ComK was found (Hamoen *et al*., 1998; Berka *et al*., 2002; Ogura *et al*., 2002). Although ComK was found to be solely acting as a transcriptional activator, we were interested if any downregulation within the competent subpopulation could be found using the more sensitive RNA‐sequencing technique and by using a different method to overcome the problem posed by the smaller competent subpopulation. With microarray studies not all genes within an operon were found differentially expressed (Hamoen *et al*., 1998; Berka *et al*., 2002; Ogura *et al*., 2002). RNA‐seq being more sensitive may confirm whether these genes are indeed differentially expressed during competence. To determine this, we physically separated the two subpopulations using fluorescence‐activated cell sorting (FACS). FACS allows for comparing the same number of cells of both subpopulations. A competence‐specific GFP reporter (P*comG‐gfp*) resulting in a fluorescent competent subpopulation was used to distinguish the competent from the non‐competent subpopulation (Smits *et al*., 2005). Separation and subsequent comparison of the two subpopulations results in a more natural situation than is created when using knock‐outs, as all regulatory mechanisms remain intact. This may allow for better detection of significant downregulation in the competent subpopulation. Recently, the expression and function of non‐coding RNAs (ncRNAs) in *B. subtilis* has gained substantial interest (Mars *et al*., 2016). Strain 168 harbours a large number of ncRNAs (Irnov *et al*., 2010; Nicolas *et al*., 2012). However, little is known about the expression of ncRNAs in the different *B. subtilis* subpopulations during competence. We were curious if differential expression of ncRNAs occurs, and if these ncRNAs could be regulated by ComK. In order to determine whether post‐transcriptional regulation occurs during competence, we also used LC–MS/MS to investigate protein levels between the two subpopulations. We decided to investigate the role in competence of *yhfW*, which was upregulated to similar levels as known competence genes, and its neighbouring gene *yhxC*, which shares its promoter region with *yhfW* but is transcribed in the opposite direction, in more detail.

## Results

### Differential expression of protein encoding genes

**Figure 1:**
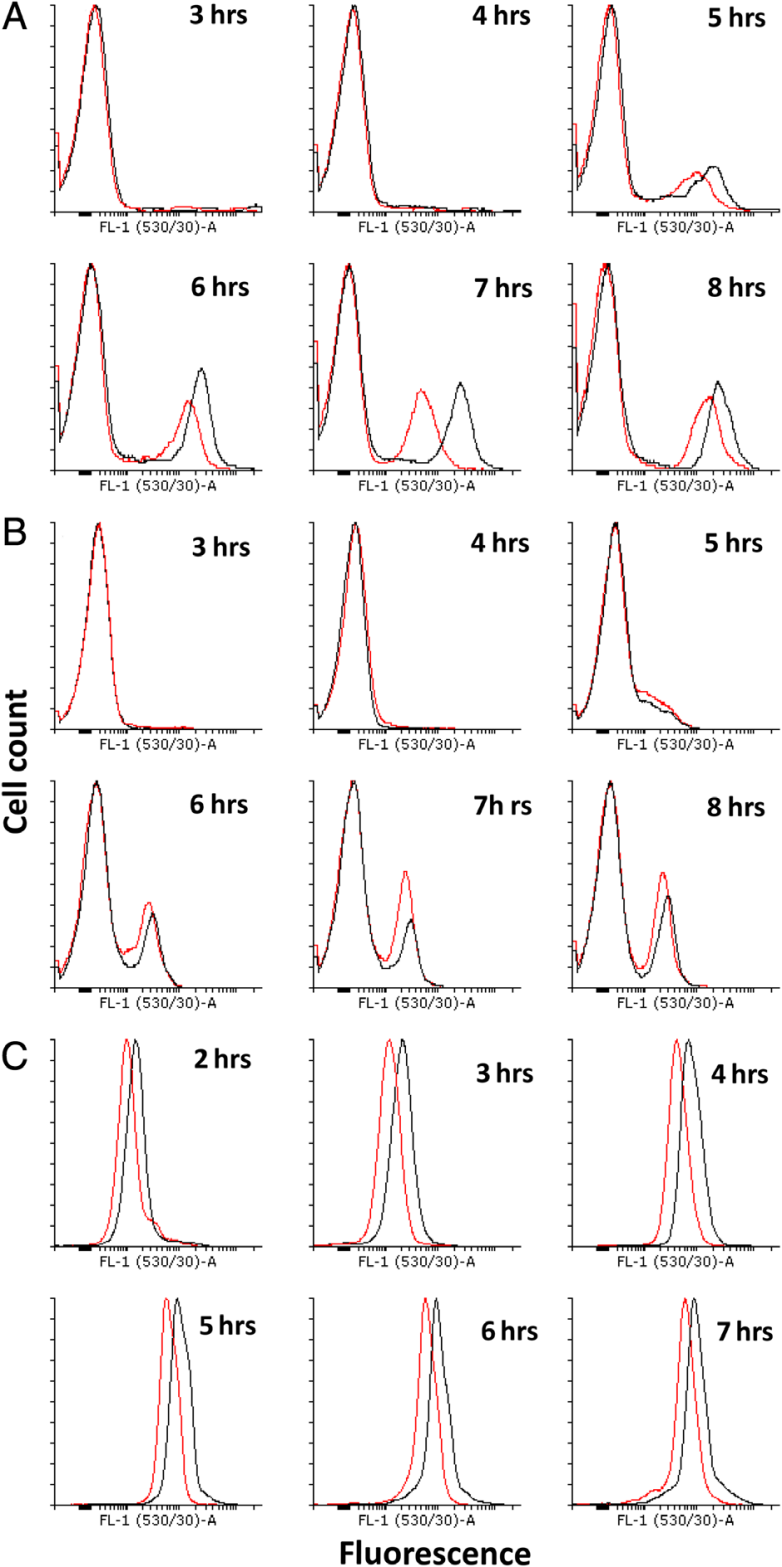
Differences in regulator expression under competence stimulating conditions. Black: control (168); red: BFA1698 (*ΔyhfW*). A. Expression of P_comG_‐*gfp* in the control (black) and the *ΔyhfW* mutant (red). The non‐competent subpopulation is represented in the left peak, and the competent subpopulation in the right peak. The expression of *comG* in the mutant is lower than in the control, shown by a shift to the left of the right red peak. The number of cells expressing* comG* in the mutant is only slightly lower than the control, shown by the lower peak height of the red right peak. B. Expression of P_comK_‐*gfp* in the control (black) and the* ΔyhfW* mutant (red). The non‐competent subpopulation is represented in the left peak, and the competent subpopulation in the right peak. The expression of *comK* in the mutant is lower, as the right red peak is shifted towards the left, but the total number of cells expressing *comK* is increased as the height of the red peak is higher. C. Expression of P_srfA_‐*gfp*. The *yhfW* mutant has lower expression of *srfA*, as the red peak is shifted towards the left. [Color figure can be viewed at wileyonlinelibrary.com]

*Bacillus subtilis* 168 P*comG‐gfp* was grown in competence‐inducing medium. The type of competence medium, type of flask and shaking conditions (oxygen availability) affect the timing of competence. Under the conditions described, cells become competent after 5 h of growth and transformability is highest during a 2 h window. Samples were taken early in the competence state at 5.5 h and at a later stage at 6.5 h in order to gain insight into the progression of competence. Cells were preserved using 2 M sodium chloride to prevent degradation of RNA before FACS and sorted into 4 M NaCl due to dilution taking place during sorting (Brown and Smith, 2009; Nilsson *et al*., 2014). The suitability of NaCl as preserving agent for preventing RNA degradation in *B. subtilis* was confirmed by comparison with flash freezing in liquid nitrogen [Supporting Information S1(Sheet)A and (Sheet)B]. We subsequently compared the transcriptomes of the competent subpopulations with those of the non‐competent subpopulations at both time points. To exclude a difference in sporulation initiation under these conditions, we specifically screened for expression of sporulation sigma factors. We did not observe a significant difference between the two subpopulations with respect to the expression of *sigE*, *sigF*, *sigG* and *sigK* and their regulons (Supporting Information S3 and Figure 1). Transcriptome data analysis of the two subpopulations was performed using T‐Rex (de Jong *et al*., 2015). A total of 156 genes were found differentially expressed when comparing the competent and non‐competent subpopulations at 5.5 h (Supporting Information S1C) and 130 genes at 6.5 h (Supporting Information S1D), when using a cut off value of twofold and maximal *P*‐value of 0.05 [EdgeR trimmed‐median mean method (TMM) normalization]. The expression levels represented as RPKM can be found in the Supporting Information S2. Our results are in accordance with previous studies with regard to the core ComK regulon (Berka *et al*., 2002; Hamoen *et al*., 2002; Ogura *et al*., 2002). Some of the genes found differentially expressed previously were not found in our results. In total, we found 90 differentially expressed genes at time point one that were not found differentially expressed in microarray studies (Table 1). Some of these genes such as *phrH*, *ccpB*, *maa* and *ybzI* are part of operons of which other genes were found differentially expressed (Berka *et al*., 2002; Hamoen *et al*., 2002; Ogura *et al*., 2002). One of the differentially expressed genes that was not picked up by microarray, and had a change of expression similar to that of the known competence genes *comFB* and *comFC*, was *yhfW*. The levels of *yhfW* in the competent subpopulation were a factor 100 lower than for *comFB* and *comFC* (Supporting Information S2). We also found several significantly downregulated genes, primarily at the first time point, with *jag* being the only gene down regulated at both time points. Two of the downregulated genes, i.e. *ywdK* and *degS* had not been previously identified as differentially expressed. Four of the downregulated genes in this study were found upregulated by Berka and co‐workers and two by Hamoen and co‐workers (Berka *et al*., 2002; Hamoen *et al*., 2002). These were *degU*, *sigA*, *jag* and *lipL* (*ywfL*). None of these genes contain a K‐box in the promoter region. Deletion of *jag*, the only gene found downregulated at both time‐points, did not result in a change in competence (data not shown). We also compared the competent subpopulation at time point one with the competent subpopulation at time point two and the non‐competent subpopulation at time point one with the non‐competent subpopulation at time point two. The results of this analysis reveal primarily higher expression of amino acid production genes at the first time point (Supporting Information S1E and F).

**Table 1:** Differentially expressed protein coding genes that were not found differentially expressed previously. The complete lists of differentially expressed genes for both time points can be found in the Supporting Information S1C + D.

Gene	Fold	Description
*antE*	11851.5	*dnaG* overlapping gene of unknown function
*yozZ*	7427.1	Hypothetical protein/pseudogene
*ybxH*	5965.9	Hypothetical protein
*ydzL*	5744.3	Hypothetical protein
*pyrE*	5637.2	Orotate phosphoribosyltransferase
*yflD*	5461.3	Hypothetical
*ykzB*	5081.4	Hypothetical protein
*sspD*	5012.5	Small acid‐soluble spore protein D
*sspM*	4762.8	Small acid‐soluble spore protein M
*gerPE*	4494.1	Spore germination protein GerPE
*ydcT*	4397.7	Hypothetical protein
*gerPD*	4378.7	Spore germination protein GerPD
*sodF*	4214	Superoxide dismutase
*ydcO*	3259.5	Hypothetical protein
*ydhF*	3121	Hypothetical protein
*tcyL*	2869.1	l‐cystine transport system permease protein TcyL
*sspC*	2740.5	Small acid‐soluble spore protein C
*cotV*	1344.8	Spore coat protein V
*yhfW*	52.5	rieske 2Fe‐2S iron–sulfur protein YhfW
*phrH*	48.0	Inhibitor of regulatory cascade
*ygaK*	20.4	FAD‐linked oxidoreductase YgaK
*ccpB*	11.5	Catabolite control protein B
*yvqJ*	10.0	MFS transporter
*rsoA*	8.7	Sigma‐O factor regulatory protein RsoA
*ydeB*	7.6	Transcription factor YdeB
*groEL*	7.5	60 kDa chaperonin
*clpE*	7.1	ATP‐dependent Clp protease ATP‐binding subunit ClpE
*maa*	7.0	Maltose o‐acetyltransferase
*ybzI*	6.1	Hypothetical protein
*gid*	6.0	Methylenetetrahydrofolate‐‐tRNA‐(uracil‐5‐)‐methyltransferase TrmFO
*sacB*	5.6	Levansucrase
*yeeI*	5.1	Transcriptional regulator
*ypzG*	5.0	Hypothetical protein
*ybdJ*	5.0	Transcriptional regulator
*sdpC*	4.7	Killing factor SdpC
*yjcM*	4.4	Hypothetical protein
*yopL*	4.4	Hypothetical protein
*ydzE*	4.2	Permease
*radA*	4.1	DNA repair protein RadA
*ymzE/2*	3.9	Pseudogene
*holA*	3.9	DNA polymerase III, delta subunit
*eglS*	3.9	Endoglucanase
*sigO*	3.9	RNA polymerase sigma factor SigO
*yoqW*	3.8	Hypothetical protein
*yjiA*	3.7	Hypothetical protein
*parA*	3.6	Sporulation initiation inhibitor protein Soj
*mta*	3.5	HTH‐type transcriptional activator mta
*yocI*	3.4	ATP‐dependent DNA helicase RecQ
*parB*	3.4	Stage 0 sporulation protein J
*ycgP*	3.3	Hypothetical protein
*ytzJ*	3.3	Hypothetical protein
*ftsR*	3.2	LysR family transcriptional regulator
*hrcA*	2.8	Heat‐inducible transcription repressor HrcA
*yeeK*	2.8	Spore coat protein YeeK
*mcsA*	2.7	Hypothetical protein
*licT*	2.7	Transcription antiterminator LicT
*bpr*	2.7	Bacillopeptidase F
*gidA*	2.5	tRNA uridine 5‐carboxymethylaminomethyl modification enzyme MnmG
*mcsB*	2.4	ATP:guanido phosphotransferase YacI
*yddT*	2.3	Hypothetical protein
*comN*	2.2	Post‐transcriptional regulator
*aroD*	2.2	3‐dehydroquinate dehydratase
*degS*	−2.3	Signal transduction histidine‐protein kinase/phosphatase DegS
*sigA*	−2.4	RNA polymerase sigma factor RpoD
*ywdK*	−2.5	Hypothetical protein
*degU*	−2.7	Transcriptional regulatory protein DegU
*jag*	−2.8	Protein* jag*
*lipL*	−3.0	Octanoyl‐[GcvH]:protein N‐octanoyltransferase
*ylaD*	−1630.8	Anti‐sigma‐YlaC factor YlaD
*ynzL*	−1845.5	Hypothetical protein
*ydzS/1*	−4271.6	Pseudogene

### Expression patterns of non‐coding RNAs

As little is known about the expression of ncRNAs during competence, we decided to look at their expression patterns under competence conditions. We found a total of 37 elements, 17 of which are antisense RNAs (Table 2 and Supporting Information S1G and H). The previously found upregulated genes *degU*, *sigA*, *jag* and *lipL* were found to have upregulated anti‐sense RNAs. We also found upregulation of S963 which is anti‐sense to *comER*. The upregulation of *comER* in microarray studies was previously suggested to be a false positive caused by upregulation of anti‐sense RNA (Hamoen *et al*., 2002). To determine whether the expression of the ncRNAs could be controlled by ComK we looked at the presence of potential K‐boxes in their respective promoter regions using Genome2D TFBS (Baerends *et al*., 2004). We found potential K‐boxes for seven of the ncRNAs within the first 100 bp upstream region and two ncRNAs with K‐boxes within the first 300 bps (Table 2). Ten ncRNAs are preceded by competence genes with K‐boxes in their respective promoter regions, and these ncRNAs read in the same direction as the competence genes. The majority of the antisense RNAs are preceded by potential K‐boxes. We did not find ncRNAs with a K‐box at the second time point that were not present at the first time point. Although we found 17 antisense RNAs, only four of the upregulated antisense RNAs have corresponding downregulated genes. These are *degU*, *lipL*, *jag* and *sigA*. S1458 is a very large antisense RNA that overlaps with four genes (*pta*, *cysl*, *lipL* and *ywfM*). S1579, i.e. the *jag* and *spoIIIJ* antisense RNA, was also upregulated in our data. Upregulated S951 is antisense to *sigA* and partially overlaps *dnaG*. The only downregulated gene not covered by an antisense RNA was *ywdK*.

**Table 2:** Differential expression of ncRNAs at the first time point. The description is taken from the study by Nicolas *et al.* (2012).

Name	Fold	Antisense	Description	K‐box	bp distance to start transcript
S963	184.6	*comER*	5'UTR of *comEA*	II‐14	31
S962	173.6	*yqzM*	Independent transcript	*comE*	
S1354	167.8	*degU*	Independent transcript	I‐13	65
S1458	166.4	*pta*	5'UTR of *hemQ*	I‐15	29
S98	121.5	*cwlJ*	5'UTR of *ycbP*	II‐14	0
S122	117.4	*bglC*	Intergenic region	*nucA*	
S125	113.2	*tlpC*	5'UTR of *hxlR*	II‐13	95
S1399	100.8		3'UTR of *ssbB*	*ssbB*	
S652	98.1	*yndK*	3' of S653	No	
S1579	96.6	*spoIIIJ*	Independent transcript	II‐15	5
S97	93	*ycbO*	3'UTR of *ycbP*	No	
S925	80.3	*yqzG*	3'UTR of *yqzE*	*comG*	
S245	43.4		Intergenic region	*rapH*	
S1357	32.3		5'UTR of *yvyE*	No	
S1575	27.9		5'UTR of *rpsF*	No	
S401	26	*yjzB*	Intergenic region	Med	
S1175	24.2		5'UTR of *mntA*	II‐15	51
S1353	22.3		Intergenic region	*comF*	
S366	22.1	*yhxD*	Intergenic region	*comK*	
S655	21.5	*yndL*	5' of S653	No	
S367	17.3	*yhxD*	Intergenic region	*comK*	
S951	16.1	*sigA*	Independent transcript	No	
S876	11.3	*aroC*	3'’UTR of *serA*	No	
S1278	10.6		5'UTR of *oxdC*	No	
S583	10.2		5'UTR of *topA*	I‐13	275
S653	9.6		independent transcript	No	
S208	8.9		5'UTR of *groES*	No	
S209	8.3		3'UTR of *groEL*	No	
S967	5.8		3'UTR of *sda*	No	
S959	4.6		intergenic region	No	
S30	4		5'UTR of *sspF*	No	
S1577	3.2		intergenic region	*trmE*	256
S174	3.1		3'UTR of *yddM*	No	
S515	2.8		Intergenic region	No	
S296	−2.9		5'UTR of *yfhP*	No	
S488	−5.4		5'UTR of *ykvA*	No	

The second last column indicates if the ncRNA has a K‐box predicted by Genome2D TFBS. The type of K‐box was manually determined according to the specifications used by Hamoen *et al*. (2002). The last column indicates the distance of the K‐box to the start of the transcript, measured from the end of the K‐box to the start codon.

### Differential protein levels between the competent and non‐competent subpopulations

For the DNA repair/recombination genes *addA* and *addB*, no significant changes in transcription were found during competence in our or in previous studies (Berka *et al*., 2002; Hamoen *et al*., 2002; Ogura *et al*., 2002). However, they were found to affect transformation through mutation (Alonso *et al*., 1993). Others, such as *sbcC* and *noc*, were found differentially expressed in only one of the three micro‐array studies (Ogura *et al*., 2002). Because regulation can also occur at the post‐transcriptional level, it is possible that they have different protein levels in the competent subpopulation. We decided to investigate protein levels in the competent and non‐competent subpopulations to determine whether these proteins do indeed have different levels. Other proteins may also have different levels in the competent subpopulation but no corresponding change in RNA levels. For this experiment, *B. subtilis* 168 cells, sampled at 5.5 and 6.5 h, were sorted by FACS onto a filter manifold system. The filters were collected and stored at −80°C. Samples were digested and analysed by LC–MS/MS. At the first time point, we found 53 proteins to be differentially expressed, six of which were downregulated in the competent subpopulation (Table 3 and Supporting Information S1I). The second time point had 94 differentially expressed proteins, 20 of which were downregulated in the competent fraction (Supporting Information S1J). Twenty‐three of the proteins found in the first time point and 20 of the proteins found in the second time point were also found in the RNA‐seq data. None of the genes found downregulated in the RNA‐seq data were found to have lower protein levels. None of the downregulated genes found in the protein data were found in the RNA‐seq data. Most of the proteins with lower levels in the competent subpopulations are involved in metabolism, with a few unknown genes at the second time point. As expected, some of the proteins for which the corresponding gene was found differentially expressed in only one of the microarray studies were found to have different amounts in our proteomics data. These proteins were Noc, SbcC and SbcD. For some of the proteins for which we found differential levels, such as MinD and Noc, their corresponding genes are part of an operon, in which other genes were found differentially expressed at the RNA level. Nucleoid occlusion protein gene *noc* is part of the *trmE* operon of which *thdF*, *gidA* and *gidB* were also upregulated at the RNA level. The gene of cell division inhibitor MinD lies in an operon with *mreB*, *radC* and *maf*. The deoxyribonuclease subunits *addA* and *addB* were found to be involved in transformation through mutation analyses (Alonso *et al*., 1993; Fernández *et al*., 2000). However, they were not found differentially expressed on either the protein or RNA level in our or the microarray studies. These genes form an operon with the DNA exonucleases *sbcC*, and *sbcD* and the HNH like nuclease *hlpB*. Only *sbcC* was found differentially expressed at the RNA level in one of the replicates of Ogura and co‐workers (Ogura *et al*., 2002). We also find higher levels of the zinc transporter ZosA, which affects competence (Ogura, 2011). Other interesting proteins with higher levels in the competent subpopulation are the fatty acid biosynthesis proteins FabHA and FabF (5.5 h), and FloT, which is involved in regulation of membrane fluidity and the formation of lipid rafts. In the same operon as the known competence gene *coiA* lies *pepF*, for which we found higher protein levels in the competent subpopulation.

**Table 3:** Differential protein levels at time point 1. The data for both time points can be found in the Supporting Information S1I and J.

Protein	LogFC	Description
ComEB	6.48	Late competence protein required for DNA binding and uptake
NucA	6.24	catalyses DNA cleavage during transformation
Nin	5.69	Inhibitor of the DNA degrading activity of NucA
RecA	4.17	Homologous recombination
SsbA	4.14	Single‐strand DNA‐binding protein
YyaF	3.86	GTP‐binding protein/GTPase
FlgL	3.11	Flagellar hook‐associated protein 3 (HAP3)
FliW	2.78	Checkpoint protein for hag expression, CsrA anatagonist
YdeE	2.64	Similar to transcriptional regulator (AraC family)
YvrP	2.44	Unknown
TrmFO	2.35	tRNA:m(5)U‐54 methyltransferase, glucose‐inhibited division protein
Maa	1.96	Maltose O‐acetyltransferase
SucD	1.79	Succinyl‐CoA synthetase (alpha subunit)
SucC	1.7	Succinyl‐CoA synthetase (beta subunit)
YlbA	1.67	Unknown
FloT	1.59	Involved in the control of membrane fluidity
TagT	1.57	Phosphotransferase, attachment of anionic polymers to peptidoglycan
Noc	1.46	Spatial regulator of cell division to protect the nucleoid
BdbD	1.41	Required for the formation of thiol disulfide bonds in ComGC
Ffh	1.4	Signal recognition particle (SRP) component
Spo0J	1.36	Chromosome positioning near the pole, antagonist of Soj
SipW	1.25	Signal peptidase I
GidA	1.24	Glucose‐inhibited division protein
ThdF	1.23	GTP‐binding protein, putative tRNA modification GTPase
YckB	1.23	Similar to amino acid ABC transporter (binding protein)
GrpE	1.21	Heat‐shock protein (activation of DnaK)
YfmM	1.17	Similar to ABC transporter (ATP‐binding protein)
YwfH	1.14	Short‐chain reductase
SbcD	1.12	Exonuclease SbcD homologue
MurB	1.1	UDP‐N‐acetylenolpyruvoylglucosamine reductase
YdgI	1.05	Similar to NADH dehydrogenase
YvbJ	1.01	Unknown
ClpY	1.01	Two‐component ATP‐dependent protease, ATPase subunit
HemQ	0.99	Heme‐binding protein, essential for heme biosynthesis
FabHA	0.98	Beta‐ketoacyl‐acyl carrier protein synthase III
ZosA	0.95	Zinc transporter
HprT	0.93	Hypoxanthine phosphoribosyltransferase
SwrC	0.91	Similar to acriflavin resistance protein
GroEL	0.9	Chaperonin and co‐repressor for HrcA
FabF	0.89	Involved in the control of membrane fluidity
YtsJ	0.83	Malic enzyme
MinD	0.81	cell‐division inhibitor (septum placement)
SbcC	0.79	DNA exonuclease
PepF	0.77	Oligoendopeptidase
DltC	0.76	d‐alanine carrier protein
YtwF	0.7	Unknown
YqaP	0.68	Unknown
HisD	−0.8	Histidinol dehydrogenase
PyrAA	−0.86	Carbamoyl‐phosphate synthetase (glutaminase subunit)
PheS	−0.99	Phenylalanyl‐tRNA synthetase (alpha subunit)
HisG	−1.12	ATP phosphoribosyltransferase
GudB	−1.23	Trigger enzyme: glutamate dehydrogenase
AtpF	−0.83	ATP synthase (subunit b)

### Investigations into *yhfW* and *yhxC*

**Figure 2:**
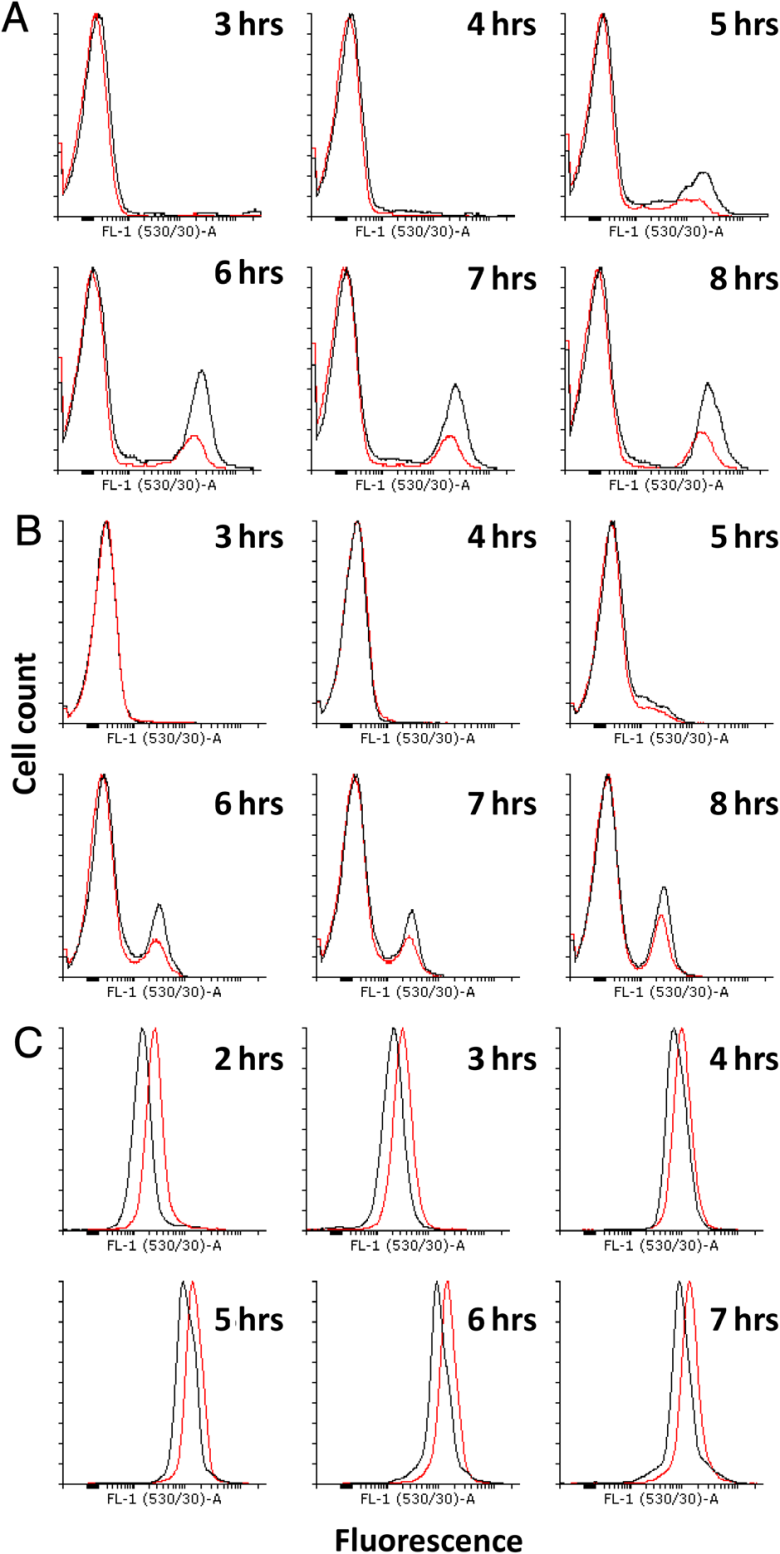
Differences in regulator expression under competence stimulating conditions. Black: control Red: BFA1701 (*ΔyhxC*). A. Expression of P_comG_‐*gfp* in the control (black) and the *ΔyhxC *mutant (red). The non‐competent subpopulation is represented in the left peak, and the competent subpopulation in the right peak. The expression of *comG* in competent cells of the mutant is the same as in the control, as the black and red right peaks are at nearly the same position on the X‐axis. The number of cells expressing *comG* in the mutant however is lower than the control, shown by the much lower peak height of the red right peak. B. Expression of P_comK_‐*gfp* in the control (black) and the *ΔyhxC* mutant (red). The non‐competent subpopulation is represented in the left peak, and the competent subpopulation in the right peak. The expression of *comK* in competent cells in the mutant is the same as for the control as there is no shift in the right red peak compared to the right black peak. The total number of cells expressing *comK *is decreased as the height of the red peak is much lower than the black peak. C. Expression of P_srfA_‐*gfp*. The *yhxC* mutant has higher expression of *srfA*, as the red peak is shifted towards the right. [Color figure can be viewed at wileyonlinelibrary.com]

**Figure 3:**
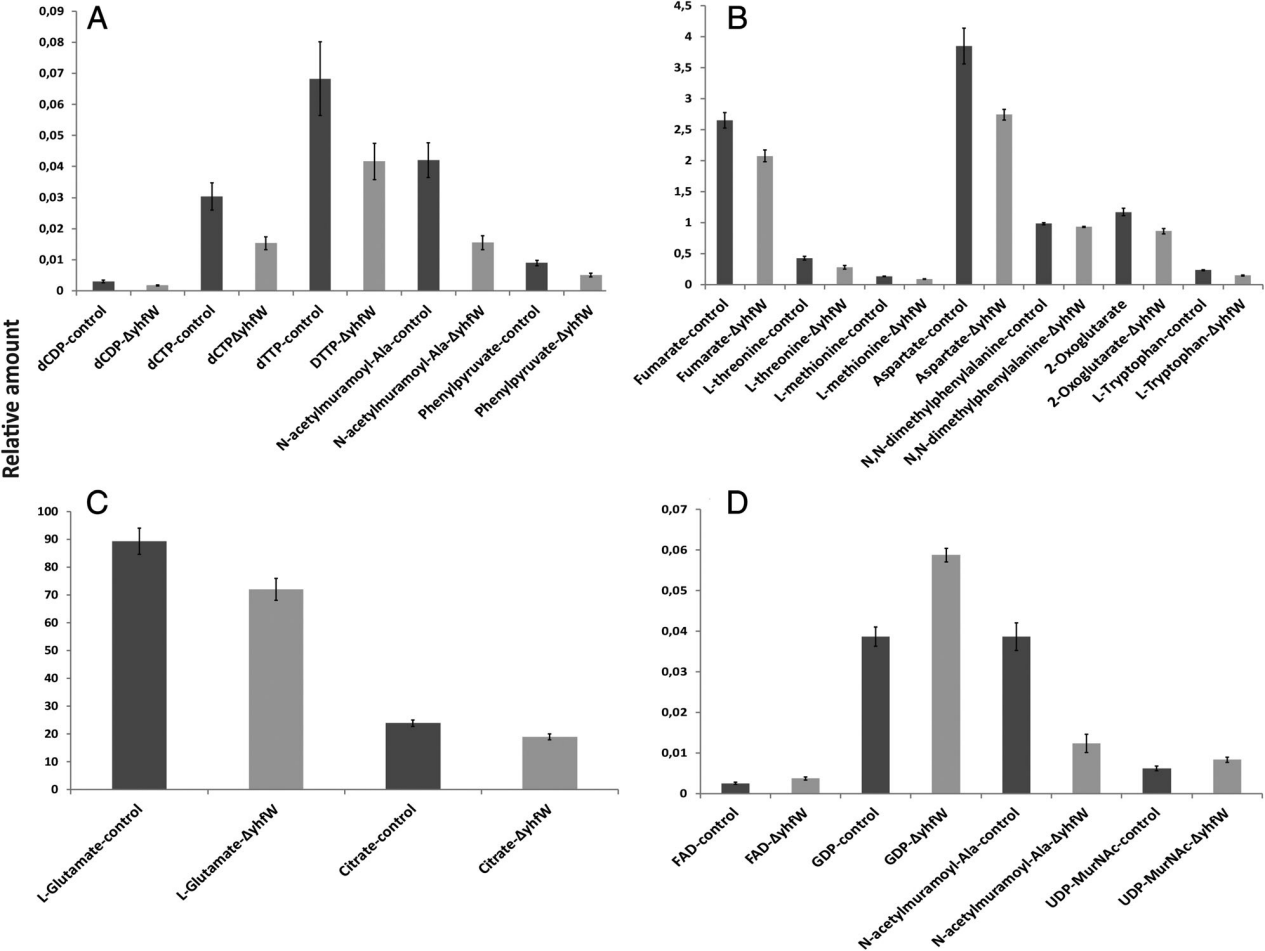
Relative difference in metabolites *ΔyhfW* vs control. A–C. Statistically significant differences between *ΔyhfW* (light grey) and the control (dark grey) under competence conditions after 6 h determined by LC–MS. D. Statistical significant differences between *ΔyhfW* and the control under competence conditions after 7 h determined by LC–MS. [Color figure can be viewed at wileyonlinelibrary.com]

**Figure 4:**
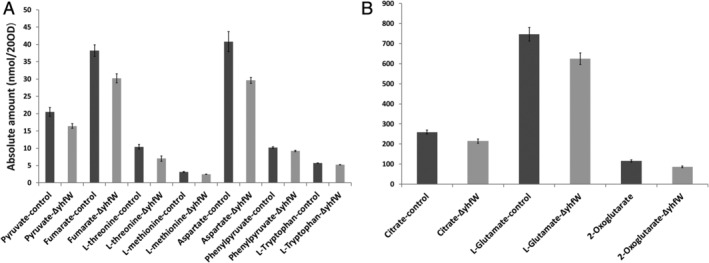
Absolute difference in metabolites *ΔyhfW* versus control. A and B. Statistically significant differences between *ΔyhfW* (light grey) and the control (dark grey) under competence conditions after 6 h determined by GC–MS.

**Figure 5:**
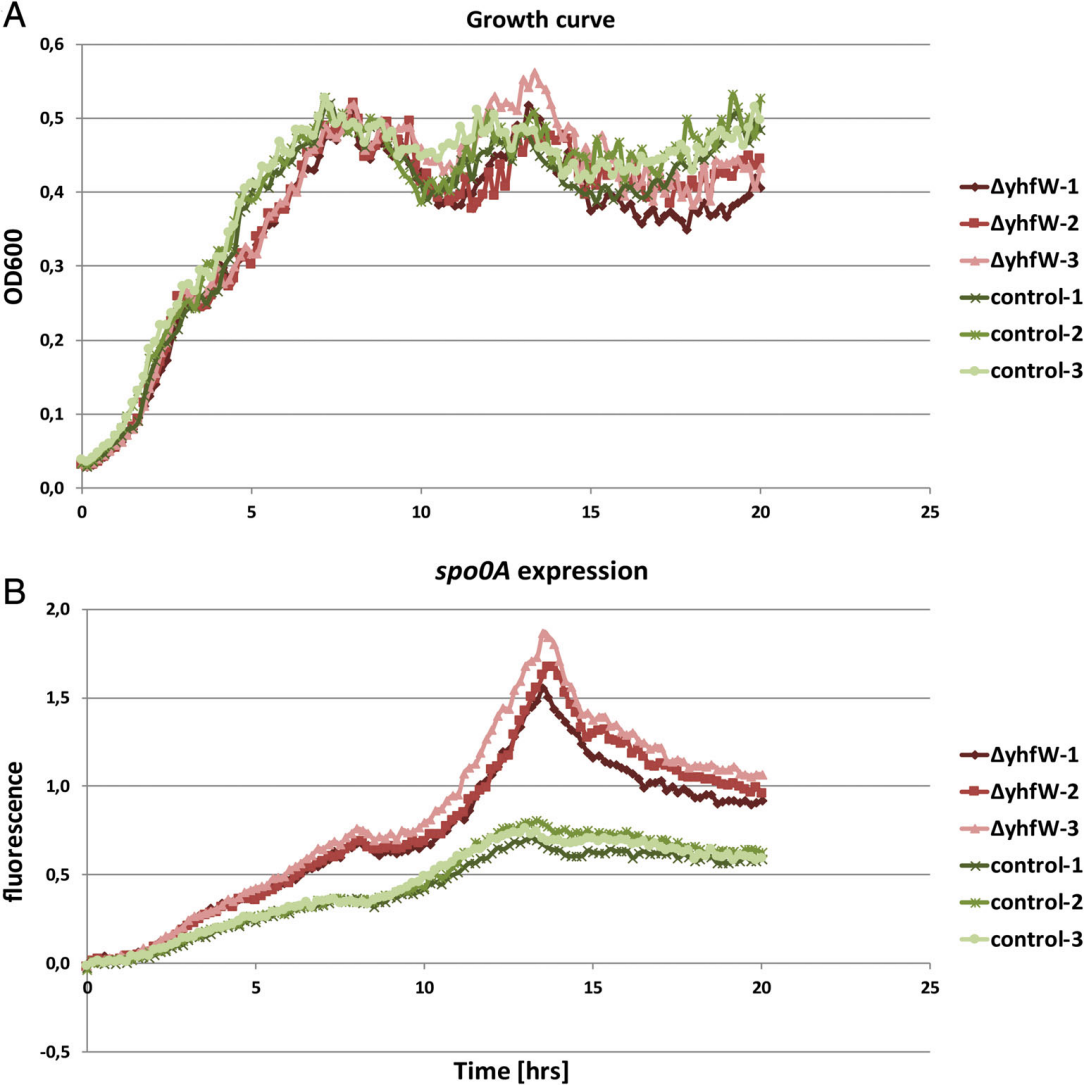
Difference of expression of spo0A in ΔyhfW and control under sporulation conditions. A. Growth curve. Green control, red ΔyhfW. B. Expression of spo0A. Green control, red ΔyhfW. [Color figure can be viewed at wileyonlinelibrary.com]

**Figure 6:**
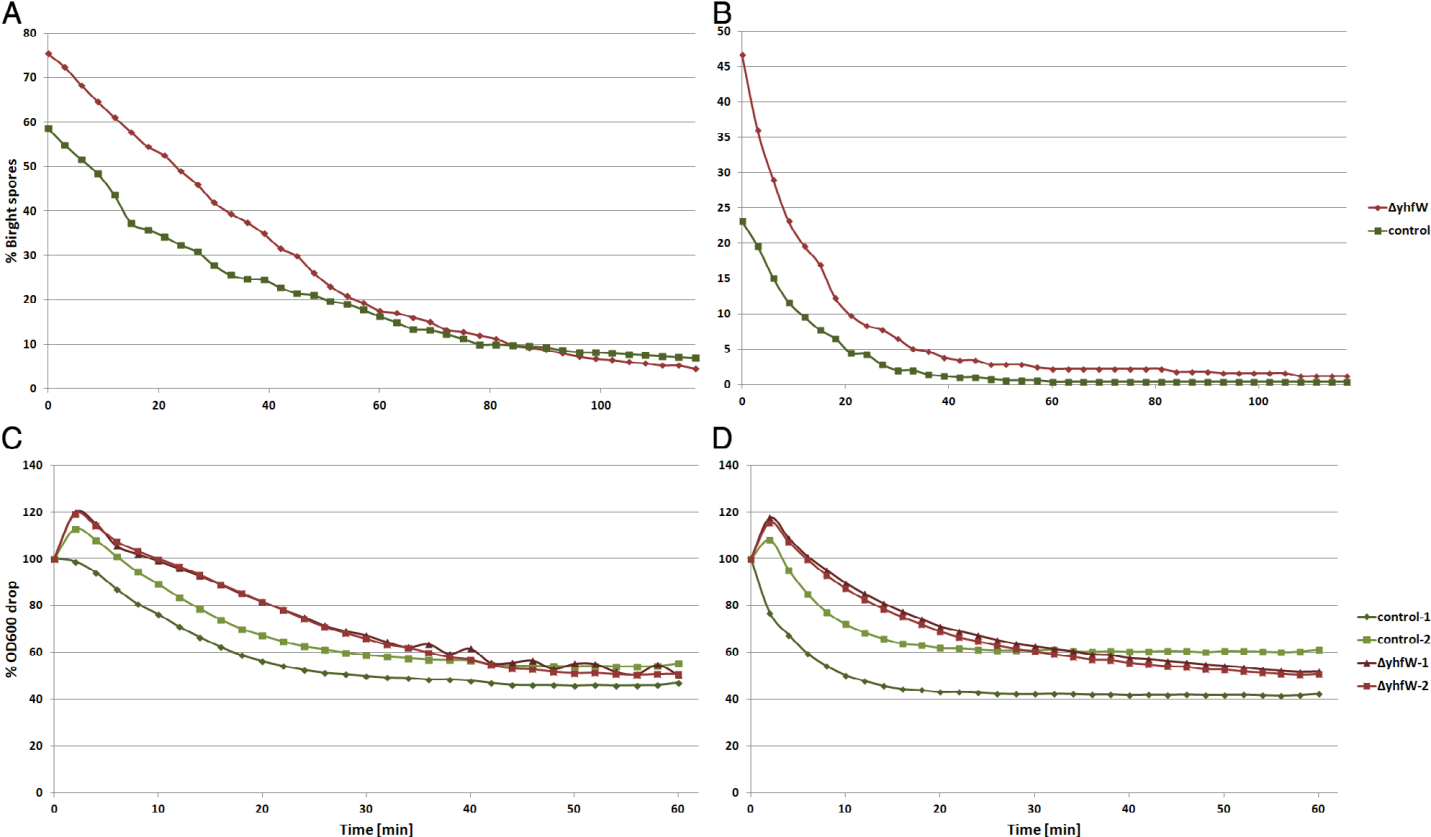
Differences in germination between *ΔyhfW* and the control 168. A. Germination followed by time‐lapse microscopy. The graph represent the percentage of bright spores of *ΔyhfW* (red) and the control 168 (green) of spores that were not heat treated before the start of the experiment. The percentage of bright spores decreases more slowly for the mutant *ΔyhfW* than for the control, representing slower germination. B. Germination of heat‐treated spores followed by Time‐Lapse microscopy. Red *ΔyhfW* and green control 168. As for the non‐heat treated spores. The percentage of bright spores decreases more slowly for the mutant *ΔyhfW* than for the control, representing slower germination. C. Germination of non‐heat‐treated spores followed by incubation in a plate reader. Germination of spores causes a reduction in the OD which occurs more slowly in the *yhfW* mutant (red).D. Germination of heat‐treated spores followed by incubation in a plate reader. Germination of spores causes a reduction in the OD which occurs more slowly in the *yhfW* mutant (red). [Color figure can be viewed at wileyonlinelibrary.com]

Among the newly found genes in our study, *yhfW* was upregulated to a similar level as the known competence genes *comFC* and *comEB*. Interestingly, it does not have a ComK‐binding site in its promoter region, and its expression pattern does not match other genes regulated by ComK during competence (Supporting Information S3 and Fig. 2). Despite not having a K‐box in the promoter region, we hypothesized that *yhfW* might be involved in competence and that a deletion would lead to a reduction in competence, as its fold change of expression matches that of known competence genes. We performed a FACS experiment using three biological replicates of the *yhfW* mutant and the control strain grown in competence medium. We found that deletion of *yhfW* did not lead to a strong decrease in the fraction of competent cells, but rather that the expression of *comG* was significantly reduced (Mann–Whitney test, *P* < 0.04–0.001) (Fig.1A). To determine how YhfW might be affecting competence we looked at the effect of absence of YhfW on the expression of known competence regulators. We therefore tested the expression of *comK*, *srfA* and *spo0A* using three biological replicates of Δ*yhfW* and the control strain (168) (Fig. 1B and C; Supporting Information S3; Fig. 4). In the *yhfW* mutant, the *comK* expressing population was larger, but the intensity of its expression was slightly reduced. This difference was statistically significant before full formation of the competent and non‐competent subpopulations. Expression of *srfA* was also reduced in the mutant, although only statistically significant at 2 h. Expression of *spo0A* was lower in the mutant, but this effect is not statistically significant (Supporting Information S3 and Fig. 4). The expression pattern of *yhfW* is nearly identical to that of its neighbour *yhxC*, which is transcribed in the opposite direction and shares the promoter region. Both genes also share a number of predicted regulator binding sites (Supporting Information S3 and Table 1); however, the expression of *yhfW* and *yhxC* does not match other genes in these regulons during competence (Supporting Information S3 and Fig. 3). We therefore decided to also investigate the effect of inactivation of *yhxC* using three biological replicates on competence. In the absence of *yhxC*, the fraction of competent cells was significantly reduced by approximately a factor of 2 (*P* < 0.001 Mann–Whitney test) (Fig. 2A). In contrast to Δ*yhfW*, the expression of *comK* was reduced in the *yhxC* mutant, and again this difference was only statistically significant before maximum competence was achieved (Fig. 2B). The expression of *srfA* was increased in Δ*yhxC*and was statistically significant at 2, 3 and 5 h (Fig. 2C). The expression of *spo0A* was slightly lower, but as for *yhfW* not statistically significant (Supporting Information S3 and Fig. 5). To determine whether the *yhfW* and *yhxC* strains are deficient in transformation, a transformation assay using three biological replicates of Δ*yhfW*, Δ*yhxC* and the control was performed. We investigated the transformability using three types of donor DNA, the replicative plasmid pNZ8048, the integrative plasmid pDR111 and genomic DNA containing an *amyE*::P_*hyspank*_‐*spec* construct. The transformation efficiency per 1 μg of DNA was determined by comparing colony forming units (CFU) on non‐selective and selective media. The transformation efficiency for the integrative plasmid pDR111 was five times lower for Δ*yhfW* and 22 times lower for Δ*yhxC*. For the replicative plasmid, pNZ8048 transformation was 11 times lower for Δ*yhfW* and 22 times lower for Δ*yhxC*. Transformation with chromosomal DNA was 25 times lower for Δ*yhfW* and 106 times lower for Δ*yhxC*. The difference in transformation efficiencies between the three strains for each of the donor DNA types was statistically significant (Kruskal‐Wallis test) (Supporting Information S3 and Fig. 6). Overall transformation efficiency is significantly higher for the control compared to the Δ*yhfW* and Δ*yhxC* strains (Kruskal‐Wallis test) (Supporting Information S3 and Fig. 6).

### Effect of *yhfW* inactivation on the metabolome

Both YhfW and YhxC are predicted oxidoreductases of unknown function. YhfW is predicted to be a FAD‐linked oxidoreductase and contains a Rieske 2Fe‐2S domain at the C‐terminus as indicated by the InterPro functional analysis tool. YhxC belongs to the short‐chain dehydrogenase (SDR_c1) family of proteins and shows similarity to FabG and harbours 3‐oxo‐ACP reductase domains (NCBI‐pBLAST). Because YhfW is predicted to be an enzyme, we decided to determine whether inactivation of this gene would have an effect on the metabolome under competence conditions. A growth curve was determined to inspect possible differences in growth between the mutant and the control. No changes in growth rate were found for the mutant (S1‐P). Samples of four biological replicates were taken when maximum *comG‐gfp* expression was achieved; for this experiment, that time point was 6–7 h after dilution of the overnight culture. The metabolomics experiment was performed as described before (Meyer *et al*., 2013). The intracellular metabolome revealed differences in metabolite levels between the control and Δ*yhfW* (Fig. 3). At 6 h, there was a statistically significant difference in tricarboxylic acid cycle metabolites (TCA cycle), such as fumarate, 2‐oxoglutarate, and citrate. There were also significant decreases in free amino acids and amino acid intermediates such as l‐threonine, phenylpyruvate, l‐methionine, l‐tryptophan, l‐aspartate and l‐glutamate (Figs. 3 and 4 and Table 4). Other significant changes were found in dCTP an dTTP as well as the cell‐wall metabolite N‐acetyl muramoyl‐Ala. At 7 h, fewer significant differences in metabolites were found. N‐acetyl muramoyl‐Ala was significantly decreased in the mutant, whereas UDP‐MurNac, GDP and FAD were significantly increased in the mutant (Fig. 3F). Because binding sites for the regulators CcpC, CitT, CtsR and GltR were predicted by Genome2D‐TFBS to reside in the promoter region of *yhfW* and *yhxC* (Supporting Information S3 and Table 1), we examined whether the expression of *yhfW* and *yhxC* matches that of other genes within these regulons. The expression of *yhfW* and *yhxC* under competence conditions did not match those of the other genes within these regulons (Supporting Information S3).

**Table 4:** *P*‐values relative difference 6 and 7 h and absolute difference at 6 h.

Relative amount 6 h	*P* value	Absolute amount 6 h	*P* value	Relative amount 7 h	*P* value
dCDP	0.0467	Pyruvate	0.0307	FAD	0.0314
dCTP	0.0214	Fumarate	0.0093	GDP	0.000466
*dTTP	0.029	l‐threonine	0.0146	N‐acetylmuramoyl‐Ala	0.000639
*N‐acetylmuramoyl‐Ala	0.00462	*l‐methionine	0.029	UDP‐MurNAc	0.042
*Phenylpyruvate	0.029	*Aspartate	0.029		
Fumarate	0.00929	2Oxoglutarate	0.00623		
l‐threonine	0.0146	Phenylpyruvate	0.029		
l‐methionine	0.000523	l‐Glutamate	0.0325		
*Aspartate	0.029	Citrate	0.0224		
*N*,*N*‐dimethylphenylalanine	0.0383	l‐Tryptophan	0.00124		
2‐Oxoglutarate	0.00623				
l‐Glutamate	0.0318				
Citrate	0.023				
l‐Tryptophan	0.00122				

Statistics was done using a two‐tailed T‐test or Mann–Whitney test (indicated with an asterisk) on four biological replicates.

### Transcriptomic analysis of BFA1698 (Δ*yhfW*)

To determine whether the changes in metabolites correspond to changes in expression of genes encoding amino acid biosynthesis and TCA cycle enzymes in the mutant, we performed RNA‐seq on samples harvested at the same time in the same experiment as those used for the metabolomics experiment. Although there were quite a few metabolites with significantly changed levels, we only found 17 differentially expressed genes in the RNA‐seq data (Table 5). None of the genes found are known amino acid biosynthesis or TCA cycle genes. We did find upregulation of NAD biosynthesis genes *nadA*, *nadB* and *nadC*. The expression of the three NAD synthesis genes is low under competence conditions in wild‐type *B. subtilis* (Supporting Information S2). Interestingly, we do not observe a significant increase in the levels of NAD nor in the levels of NADP in the metabolomics data (Supporting Information S1 N). We also found upregulation of the Na+/H+ antiporter *nhaC*. The majority of the downregulated genes have no known function, but the expression pattern of *yxeD* and *sspD* is very similar to that of *yhfW* (Nicolas *et al*., 2012).

**Table 5:** Differentially expressed genes in the ΔyhfW mutant under competence conditions. Samples for RNA‐seq were from the same experiment and were taken at the same time timepoints as the samples taken for metabolomics analysis.

Fold	Gene	Description
39.1	*nadB*	l‐aspartate oxidase
35.5	*nadC*	Nicotinate‐nucleotide diphosphorylase (carboxylating)
29.2	*nadA*	Quinolinate synthetase
11.7	*lip*	Extracellular lipase
7.3	*trnY‐Phe*	Transfer RNA‐Phe
5.5	*nhaC*	Na/H antiporter
5.2	*tyrS*	Tyrosyl‐tRNA synthetase
4.3	*yrzI*	Unknown
4	*opuCB*	Glycine betaine/carnitine/choline ABC transporter
−3.7	*ykzN*	Unknown
−6.1	*corA*	Unknown
−8.7	*ywjC*	Unknown
−11.9	*ywqJ*	Unknown
−42	*yosF*	Unknown
−79.5	*sspP*	Probable small acid‐soluble spore protein
−204.3	*yxeD*	Unknown
−334.7	*ywqI*	Unknown

### Effects of *yhfW* deletion on sporulation

As *yhfW* is primarily regulated by SigF, we decided to determine whether the absence of *yhfW* could lead to a statistically significant difference in *spo0A* expression under sporulation conditions. BFA1698 (Δ*yhfW*) was grown in chemically defined sporulation medium + alanine CDSM for 20 h. In contrast to the competence stimulating conditions, growth in CDSM + A significantly affects the expression of *spo0A*. Interestingly, the expression of *spo0A* was higher in the mutant compared to the control (Fig. 5B), whereas the expression of *spo0A* was lower in the mutant under competence stimulating conditions (Supporting Information S3 and Fig. 1). To determine whether there is an actual difference in the sporulation efficiency of Δ*yhfW*, sporulation assays were performed on three biological replicates of the control and mutant grown in CDSM + A. Sporulation efficiency was determined by determination of CFUs before and after treatment with 10% chloroform or heat treatment. The sporulation of cultures grown for 24 h in CDSM + A was low for both control (1% chloroform, 1.4% heat) and Δ*yhfW* (0.6% chloroform, 0.31% heat). Sporulation efficiency for Δ*yhfW* under these conditions is 1.8 times lower for the chloroform treatment and 4.6 times lower for the heat treatment; however, these differences were not statistically significant.

### Germination efficiencies of Δ*yhfW* and wt strains

YhfW was found to be a spore coat protein by Abhyankar and co‐workers (Abhyankar *et al*., 2015). We therefore also looked at the germination efficiency of the Δ*yhfW* strain. For this experiment, the control and Δ*yhfW* strains were grown in chemically defined sporulation medium (CDSM), and the spores were harvested after 24 h and used for germination assays. When looked at under a microscope, mature spores show up as light/bright and become dark when they germinate. Germination was determined by a time‐lapse experiment of heat treated and non‐heat‐treated spores placed on a slice of LB‐containing agarose and counting the bright versus dark spores every 2 min. Germination was also investigated by detecting the OD drop corresponding to germination, of spores incubated in LB in a Varioscan plate reader. A clear reduction in germination speed in the *yhfW* mutant was found in both experiments (Fig. 6).

## Discussion

Our results are largely in accordance with previous studies with regard to the core ComK regulon (Supporting Information S1C + D). Some of the genes found in previous studies were not found in our data. This is likely because no knock‐out mutants of *comK* and/or *mecA* were used in our experiment, and therefore both compared populations are under natural control of the relevant regulators. We found six genes that were significantly downregulated in the competent subpopulation (Table 1) and four of which have corresponding upregulated antisense RNAs (Table 2). These were *degU*, *jag*, *sigA*, and *lipL*. These genes were previously found upregulated, however, this was likely the result of the use of amplicon arrays. Because the probes in amplicon arrays constitute double stranded DNA (dsDNA) it cannot distinguish between sense and antisense DNA. Hamoen and co‐workers already determined that *comER* was one of these false positives, and, indeed, we found upregulation of the antisense *comER* RNA (S963) but not of *comER* itself (Hamoen *et al*., 2002). Lower levels of the housekeeping sigma factor *sigA* may be related to a reduced need for expression of housekeeping genes as cell division and replication are halted during competence. DegU is a regulator of competence as well as of degradative enzyme expression and biofilm formation. It regulates its own expression by binding to the *degU* promoter region (Dahl *et al*., 1992; Mäder *et al*., 2002; Veening *et al*., 2008; Ogura and Tsukahara, 2010). The samples were taken at the point of maximum competence, and downregulation of *degU* may represent the reduced need for DegU‐mediated activation of *comK* expression. The *lipL* gene that we found downregulated, and which is covered by antisense RNA S1458 is essential for lipoic acid formation. Lipoic acid is necessary for the pyruvate dehydrogenase complex of which one subunit affects Z‐ring formation (Perham, 2000; Christensen *et al*., 2011; Martin *et al*., 2011; Monahan *et al*., 2014). S1458 also covers the *pta gene*, encoding phosphotransacetylase, which has been found to affect cell division in *E. coli*. However, we did not find significant downregulation of the other genes in this operon (Maciąg‐Dorszyńska *et al*., 2012). Downregulation of genes involved in cell division may be related to the fact that cell division is halted during competence. The majority of the downregulated genes have higher levels of corresponding antisense RNAs that contain predicted K‐boxes in their promoter regions. The actual mode of action of these antisense RNAs will also have to be determined as was done for the *gdpP* asRNA by Luo and Helmann (2012). The downregulation of the corresponding gene may be a by‐product of the transcription of the anti‐sense RNA without a true phenotype resulting from their interaction (Mars *et al*., 2016). Further studies are thus required to confirm direct regulation of the antisense RNAs by ComK and their effect on their opposite genes during competence. No direct downregulation by ComK was found in our results and so far, *kre* may be the only gene directly inhibited by ComK. The expression of *kre* is repressed in competent cells, and it contains several ComK‐binding sites (Gamba *et al*., 2015).

In the proteomics data, we found a higher number of proteins differentially expressed at the second time point. This can be explained by the maturation time of proteins and/or accumulation due to their higher stability compared to RNA. Some of the proteins found to have elevated levels in the competent subpopulation are those involved in the regulation of cell division. Halting of cell division and replication is an important aspect of competence. Known genes affecting cell division are *maf*, *noc* and *minD* (Marston *et al*., 1998; Wu *et al*., 2009; Briley *et al*., 2011). Unlike the gene for competence cell division inhibitor *maf*; *noc* and *minD* were not differentially expressed at the RNA level. They did, however, show increased protein levels in the competent subpopulation (Table 3) aside from the before mentioned MinD and Noc. The exonucleases SbcC and SbcD also showed increased protein levels in the competent subpopulation but were not differentially expressed at the RNA level. Our results, combined with previous research, show that MinD, Noc, SbcC and SbcD are primarily regulated at the post‐transcriptional level. *addA* and *addB* of which mutants affect competence were not differentially expressed on either the transcriptional or post‐transcriptional level, indicating that their basal levels are sufficient for competence. We also found higher levels of FabHA and FabF and FloT in the competent subpopulation suggesting a difference in membrane lipid composition and fluidity. Levels of the zinc transporter ZosA were also higher. Disruptions in *zosA* have been shown to lead to a reduction in transformability by inhibiting the post‐transcriptional control of ComK (Ogura, 2011). There were higher levels of PepF in the competent subpopulation. Overexpression of *pepF* has been shown to inhibit sporulation initiation (Kanamaru *et al*., 2002).

One of our goals was to determine whether there are genes involved in competence that were not found in the previous transcriptomic studies by using a direct approach in comparing competent and non‐competent subpopulations, instead of mutants. We did indeed find upregulation of several genes that were not found differentially expressed previously, and most notably we found strong upregulation of *yhfW* (Table 1), which encodes a FAD‐dependent oxydoreductase of unknown function. YhfW is conserved among *B. subtilis* group species with a sequence identity ranging from 60% to 94% over 91% to 100% of the sequence (NCBI pBLAST). Homologues with >50% identity over >96% sequence coverage can be found in the following orders within the *Bacilli* class: *Alicyclobacillaceae*, *Bacillaceae*, *Paenibacillaceae*, *Planococcaceae*, *Sporolactobacillaceae*, *Thermoactinomycetaceae*, and the unclassified *Desulfribacillus*, *Flavobacterium thermophilum* and *Acidibacillus*. Within the phylum *Firmicutes*, the *Clostridiaceae* family also contains homologous proteins with a sequence identity of >40% and over 96% of the sequence. Interestingly, proteins with over 40% sequence identity over >96% of the sequence are also found in the phyla *Bacteriodetes* (*Flavobacteria*), *Proteobacteria* (alpha and beta), *Actinobacteria* (*Actinobacteria*) and in the Archea species *Methanosarcina* and *Methanoculleus*. In *B. subtilis*, deletion of *yhfW* reduced *comG* expression and caused a change in expression under competence conditions of the important *B. subtilis* regulators *comK* and *srfA*. Its neighbouring gene, *yhxC* also affects expression of *comG*, *comK*, and *srfA*. In contrast to *yhfW*, deletion of *yhxC* resulted in a strong decrease in the number of competent cells. Deletion of *yhfW* or *yhxC* also caused a significant reduction of transformability of *B. subtilis*. Absence of YhfW under competence conditions resulted in a significant decrease of several TCA cycle metabolites and aminoacids (Fig. 3) and upregulation of *de novo* NAD/NADH synthesis genes (Table 5). Biosynthesis of NAD in *B. subtilis* occurs from aspartate and uses fumarate or oxygen as electron acceptor for FAD reoxidation (Marinoni *et al*., 2008).

It is possible that the changes in TCA cycle and a possible resulting defect in NAD/NADH homeostasis is responsible for upregulation of NAD synthesis genes or that the upregulation of NAD synthesis disrupts NAD/NADH homeostasis. Further processing of NAD in the nicotinate and nicotinamide pathway may explain why higher levels of NAD/NADH were not detected. Upregulation of *nhaC* may be a result of internal pH disruptions due to the lower levels of amino acids and intermediates such as fumarate, 2‐oxoglutarate, aspartate, glutamate and citrate. NhaC has been found to be involved in pH homeostasis and the uptake of Na+ (Prágai *et al*., 2001). As we did not find significant changes in the expression levels of amino acid synthesis genes, it seems likely that the reduction in the levels of amino acid synthesis intermediates and amino acids is the result of a disruption in the TCA cycle.

Aside from its effect on competence, deletion of *yhfW* increased expression of *spo0A* under sporulation conditions (Fig. 4). However, sporulation efficiency was not significantly affected under the conditions tested. Different sporulation conditions however may result in a significant effect. Spo0A is only active in its phosphorylated state, and upregulation of *spo0A* alone may thus not be enough for a phenotypic effect on sporulation (Ireton *et al*., 1993; Fujita and Losick, 2005). The *yhfW* mutant did show a significant reduction in germination speed. The decrease in spore outgrowth is particularly interesting in view of the results of Abhyankar and co‐workers, who indicated YhfW as a putative spore coat protein and also found YhxC in the spore coat (Abhyankar *et al*., 2015). Although *yhfW* is regulated by SigF no other SigF‐regulated genes are differentially expressed between the two subpopulations, nor is there a difference in expression of *sigF* (Supporting Information S1C + D) (Wang *et al*., 2006).

To conclude, our data confirm that ComK is primarily a transcriptional activator and that downregulation by ComK is indirect and possibly occurs through specific ncRNAs. A small number of the known competence related factors, in particular those involved in halting cell division, are primarily regulated at the protein level rather than at the transcriptional level. The high sensitivity of RNA‐seq did indeed lead to the identification of a new gene, *yhfW*, which together with *yhxC* may play an important role in the adaptive lifestyles of *B. subtilis*.

## Experimental procedures

### Growth conditions

Strains used in this study can be found in the Supporting Information S6. Unless otherwise indicated, the following competence medium was used: 18 ml demineralized water, 2 ml 10× competence medium stock [0.615 M K_2_HPO_4_•3H_2_O, 0.385 M KH_2_PO_4_, 20% glucose, 10 ml 300 mM Tri‐Na‐citrate, 1 ml 2% ammonium ferric citrate, 1 g casein hydrolysate (oxoid), 2 g potassium glutamate], 100 μl 2 mg ml^−1^ tryptophan, 67 μl 1 M MgSO_4_ (Spizizen, 1958; Konkol *et al*., 2013). Strains were streaked out from −80 stocks on Luria Bertani (LB) agar plates with antibiotics and grown overnight at 37°C. A single colony (sc) was diluted 1000× in PBS or 1× Spizizen solution 100 μl of the sc colony solution was added to 20 ml medium in 100 ml Erlenmeyer flasks and grown at 37°C 220 rpm. Exponential/early stationary overnight cultures were diluted to an OD600 of 0.05 in 20 ml medium without antibiotics. Antibiotic concentrations used were chloramphenicol (cm) 5 μg ml^−1^, spectinomycin (sp) 50 μg ml^−1^, erythromicin (ery) 0.5 μg ml^−1^, and lincomycin 12.5 μg ml^−1^. Growth conditions in CDSM (Vasantha and Freese, 1980; Hageman *et al*., 1984) + alanine (10 mM) + tryptophan 1 mM. Strains were grown overnight at 37°C on LB agar + chloramphenicol (control) or chloramphenicol + erythromycin (BFA1698), single colonies were diluted and incubated in 2 ml LB 37°C 220 rpm in test tubes. The diluted cultures were mid‐exponential after overnight growth. The overnight cultures were diluted to OD600 0.05 in 2 ml CDSM + alanine + tryptophan and chloramphenicol (control) or chloramphenicol + erythromycin (BFA1698) in test tubes and grown to mid‐exponential growth at 37°C 220 rpm. Cultures were diluted to OD600 0.1 in 100 μl CDSM + alanine + tryptophan without antibiotics in a 96 wells plate and grown at 37°C, 240 rpm, 10 min measuring interval for 20 h in a Thermo Fisher Varioskan Lux. The remainder of the cultures was grown for 24 h after which the cultures were kept in the dark at 4°C without shaking for 4 days. Spores were harvested by centrifugation at 10 000 g and washed 3× with double distilled water. The spore crops were diluted to the same OD and heated for 10 min at 80°C and dilutions were plated on LB agar with chloramphenicol and grown overnight at 37°C. Colonies were counted and measured with ImageJ. Statistics were done in Sigmaplot using a Rank Sum Test.

### Growth conditions for RNA‐seq and proteomics

*Bacillus subtilis* 168 P*comG‐gfp* chloramphenicol resistant variant was created by Prof. Dr. Jan Willem Veening. *B. subtilis* 168 P*comG‐gfp* was grown as described in growth conditions Samples for protein analysis and RNA‐seq analysis were taken at 5.5 and 6.5 h respectively. One hour of sorting through FACS yields approximately 3 × 10^7^ GFP‐negative (non‐competent cells) and 1.5 × 10^7^ GFP‐positive (competent) cells.

### Protein sample preparation and analysis

A non‐sorted control of 4.0 × 10^6^ cells was taken. A total of four biological replicates were used for the protein analysis. Samples were sorted by BD FACS Aria onto a vacuum manifold filter system. Proteins were isolated and prepared for LC/MS–MS. The on‐filter digestion method was developed by Dr. Elrike Frenzel (Functional Microbiology Division, University of Veterinary Medicine, Vienna) in cooperation with the Functional Genomics group, University Medicine Greifswald. Details regarding the digestion and MS settings can be found in the Supporting Information S4.

### Sample preparation for RNA‐seq

To prevent degradation of RNA, the cells were preserved with 2 M NaCl in PBS before FACS and sorted in to 4 M NaCl in PBS (Brown and Smith, 2009; Nilsson *et al*., 2014). The NaCl preservation method was tested by microarray analyses (Supporting Information S1A and B). Samples were harvested at 5.5 and 6.5 h, diluted in 2 M NaCl and run through BDFACS Aria at 4°C samples were sorted into 4 M NaCl on ice. Samples were filtered using a syringe and 13 mm 0.22 μm filter and washed using TE + 20 mM sodium azide and put to liquid nitrogen. The cells on the filter were homogenized in a bead mill, and RNA was extracted as described in the study by Nicolas *et al*. (2012). Two biological replicates were sent for sequencing by Primbio on a proton pI chip without ribosomal RNA depletion. Results were analysed using T‐REx (http://genome2d.molgenrug.nl) (de Jong *et al*., 2015). Comparisons were made between competent versus non‐competent cells at T1 (5.5 h), competent versus non‐competent cells at T2 (6.5 h), competent T1 versus competent cells T2, non‐competent T1 versus non‐competent cells T2. Samples for the RNA‐seq analysis of BFA1698 were harvested and extracted as described above.

### FACS analysis of regulators in a BFA1698 Δ*yhfW* and BFA1701 Δ*yhxC* background

Three single colony replicates were inoculated and grown as described under growth conditions. Samples were analysed every hour on a BD FACS Canto machine. Data were analysed using Flowing Software 2.5.1. Statistics were performed in Sigma plot using a Rank Sum test. Test.

### Transformation assay

Three single colony biological replicates of *B. subtilis BFA1698*, *BFA1701* and the control 168 were grown in competence medium as described in growth conditions. About 400 μl of culture was in incubated with 1 μg of pDR111, pHB201 or *168 amyE::Physpank‐spec* genomic DNA and incubated for 2 h. The 100 μl of culture was spread out on selective and non‐selective LB‐agar and incubated overnight at 37°C. The transformation efficiency was calculated, and statistical analysis was performed using a Kruskal‐Wallis test.

### Sporulation assay

Three single colony biological replicates of *B. subtilis 168* and BFA1698 were diluted in PBS and inoculated in 2 ml CDSM + alanine (10 mM) and tryptophan (1 mM) and incubated at 220 rpm at 37°C overnight with the antibiotics and growth conditions as described before. Exponential overnight cultures were diluted to OD600 0.05 and grown for 24 h. For each replicate, 999 μl was taken and treated with 10% end volume chloroform. For each replicate, a control was taken and treated with 10% final volume 1× PBS. Dilutions were plated out on LB agar and the CFUs were counted after overnight incubation at 37°C and the transformation efficiency calculated. For the heat treatment, 1 ml of culture was incubated for 10 min at 80°C, and the controls were kept at room temperature. Dilutions were spread on LB agar as for the chloroform treated samples.

### Germination assay

For spore isolation, 20 ml of cultures were incubated for 24 h in CDSM as described for the sporulation assay were treated with 1.5 mg ml^−1^ lysozyme for 1 h at 37°C. Subsequently, 4% final concentration of SDS was added and the samples were incubated for 30 min at 37°C. Samples were washed four times with Milli‐Q® ultra pure water (Merck Millipore) by centrifugation 5000 g, 10 min, 4°C. Cultures were re‐suspended in 2 ml Milli‐Q®. The samples were diluted to an OD of 0.1 in 200 μl incubated in a 200 μl 96‐wells plate in a Varioskan Lux at 37°C under continuous shaking at 180 rpm. Hundred millisecond measurements at 600 nm were taken at 2 min intervals. Samples for microscopy were prepared as described previously (Veening *et al*., 2009). Time‐lapse microscopy was performed on a DeltaVision Elite microscope (GE Life Sciences). Images were taken with a 60× lens with 3 min intervals, phase contrast, exposure 0.25 s, 32%.

### Metabolomics

The strains were grown in competence medium as described under growth conditions. Details on the metabolomics method can be found in the Supporting Information S5.

### Strain construction

BFA1698 (Δ*yhfW*) and BFA1701 (Δ*yhxC*) were made using pMUTIN4 by Dr. Rob Meima. BFA1698 and BFA1701 were transformed with genomic DNA from *B. subtilis* 168 P*comg‐gfp*, *B.subtilis* 168 P*comK*‐gfp, *B. subtilis* 168 P*spo0A‐gfp*, *B. subtilis* 168 P*srfA‐gfp*. The strain list can be found in the Supporting Information S6.

## Supporting information


**Table S1**: Supporting information

**Table S2**: Supporting information

**Appendix** **S3**: Supporting information

**Appendix** **S4**: Supporting information

**Appendix** **S5**: Supporting information

**Appendix** **S6**: Supporting information
