# Supplementary material for: Analyses of competent and non‐competent subpopulations of Bacillus subtilis reveal yhfW, yhxC and ncRNAs as novel players in competence
Source: Environ Microbiol. 2020 Apr 15;22(6):2312–28. doi: 10.1111/1462-2920.15005 (PMC7317962; doi:10.1111/1462-2920.15005)
Supplement: Supplementary file 3 — Appendix S3: Supporting information [file EMI-22-2312-s003.docx]

**Fig. .1** **Expression of *yhfW*  and *yhxC* under competence conditions compared with the expression of sporulation regulators, and other genes within these regulons**. There is no difference in the expression of the sporulation regulators between the competent subpopulation (green horizontal bars) and the non-competent subpopulation (grey horizontal bars)

-T1_BR1 (no- competent subpopulation, time point 1 bio-replicate 1,), +T1_BR1 (competent subpopulation, time point 1 bio-replicate 1), -T2_BR1 (non-competent subpopulation time point 2 bio-replicate 1, +T2_BR1 (competent subpopulation, time point 2 bio-replicate 1). BR2 = bio-replicate 2

**Fig. 2** **Expression of *yhfW* and *yhxC* under competence conditions compared with the expression of competence regulators and other genes within these regulons.** There is no clear correlation in expression between *yhfW* and *yhxC* and ComK regulated genes (black dots) and DegU and RocR

regulated genes

-T1_BR1 (no- competent subpopulation, time point 1 bio-replicate 1,), +T1_BR1 (competent subpopulation, time point 1 bio-replicate 1), -T2_BR1 (non-competent subpopulation time point 2 bio-replicate 1, +T2_BR1 (competent subpopulation, time point 2 bio-replicate 1). BR2 = bio-replicate 2

**Fig. 3** **Expression of *yhfW* and *yhxC* under competence conditions compared with the expression of predicted regulators of *yhfW* and *yhxC*, and other genes within these regulons.** There is no clear correlation in expression between *yhfW*  and *yhxC* with other genes within these regulons under competence conditions.

-T1_BR1 (no- competent subpopulation, time point 1 bio-replicate 1,), +T1_BR1 (competent subpopulation, time point 1 bio-replicate 1), -T2_BR1 (non-competent subpopulation time point 2 bio-replicate 1, +T2_BR1 (competent subpopulation, time point 2 bio-replicate 1). BR2 = bio-replicate 2

**Fig. 4** **Expression of *spo0A* under competence conditions**. The expression of *Spo0A* in the *yhfW* mutant (dark grey) is lower than in the control (white), but not statistically significant.

**Fig. Fig. 4** **Expression of *spo0A* under competence conditions**. The expression of *Spo0A* in the *yhxC* mutant (dark grey) is lower than in the control (white), but not statistically significant.





**Fig. 6 Transformation efficiency.**  The transformation efficiency of ΔyhfW (BFA1689), ΔyhxC (BFA1701) and the control 168 was determined for pDR111 (integrative plasmid), pHB201 (replicating plasmid) and gDNA (genomic DNA). The difference in transformation efficiencies between the three strains for each of the donor DNA types was statistically significant (Kruskal-Wallis test) Overall transformation efficiency is significantly higher for the control compared to the *ΔyhfW* and *ΔyhxC* strains. (Kruskal-Wallis test)

| **Predicted regulator** | ***yhfW*** | ***yhxC*** |
| --- | --- | --- |
| CcpC |  |  |
| CitT |  |  |
| CtsR |  |  |
| DegU |  |  |
| GltR |  |  |
| RocR |  |  |
| Xre |  |  |
| Zur |  |  |

**Table 1. Predicted regulator binding sites**

Regulator binding sites in the 300bp promoter region of *yhfW* and *yhxC.* All were predicted by Genome2D TFBS search. <http://genome2d.molgenrug.nl/>
