# Supplementary material for: Analyses of competent and non‐competent subpopulations of Bacillus subtilis reveal yhfW, yhxC and ncRNAs as novel players in competence
Source: Environ Microbiol. 2020 Apr 15;22(6):2312–28. doi: 10.1111/1462-2920.15005 (PMC7317962; doi:10.1111/1462-2920.15005)
Supplement: Supplementary file 4 — Appendix S4: Supporting information [file EMI-22-2312-s004.docx]

# Materials and Methods

The on-filter digestion method was adapted from Pförtner *et al* 2013 by Dr. Elrike Frenzel (Functional Microbiology Division, University of Veterinary Medicine, Vienna) in cooperation with the Functional genomics group, Ernst-Moritz-Arndt University, Greifswald (Pförtner *et al.*, 2013)

# Protein digestion and desalting

After cells were sorted onto a filter, cell disruption was performed on the filter by adding 200 ng of Lysozyme (Sigma–Aldrich) and 20 µl of 20 mM ammonium bicarbonate (ABC) and incubated for 30 min at 37°C. 700 ng of freshly prepared Trypsin (Promega, Madison, WI, USA) and RapiGest (Waters Corp., Milford, MA, USA) to a final concentration of 0.1% was added to enhance trypsin digestion. Trypsin digestion was stopped by adding 1% final concentration of tri-flouro acetic acid (TFA) and shaking in a mixer for 30 min at 37°C. Adding TFA also degrades the detergent RapiGest. Digested peptides were then desalted to remove the impurities that might interfere with MS analysis.

For desalting, peptides were purified and desalted using C18 resin-ZipTip® pipette tips (Millipore). ZipTip columns were equilibrated by serial pipetting of 100% ACN; 80% ACN in 1% AA; 50% ACN in 1% AA; 30% ACN in 1% AA, and 1% AA. Then the peptides were loaded onto the ZipTip by pipetting 20 times. ZipTip was washed 5 times with 1% AA and peptides were eluted in 50% ACN+1% AA and 80% ACN+1% AA. Eluates were pooled and ACN was removed by vacuum centrifuge concentrator (Eppendorf). Peptides were dissolved in Buffer A (2% ACN, 0.1% AA) to a final concentration of 100 ng/µL and stored at -20°C until MS measurement.

# LC-MS/MS acquisition

LC-MS/MS was performed by an LTQ-Velos-Orbitrap-MS (Thermo Scientific) coupled with Nano-Acquity UPLC system (Waters, Milford, MA, USA). For liquid chromatography, peptide mixtures were loaded onto a trap column (C18, 2cm length, 180 µm i.d, 5 µm particle size, Waters) and further separated on a reverse-phase analytical column (C18, 10cm length, 100 µm i.d., 1.7 µm particle size, Waters) with a flow rate of 400nL/min. Peptides were eluted with a linear gradient of 1-5% buffer B in 2 min, 5-25% B in 63 min, 25-60 % in 25 min, and 60-99% B in 2 min (buffer A-2% ACN in 0.1% acetic acid, buffer B- ACN in 0.1% acetic acid). The MS was operated in the data-dependent mode to automatically switch 300 to 2,000) were acquired in the Orbitrap with resolution R = 35,000 at m/z 400 (after accumulation to a target of 1,000,000 charges in the LTQ) allowed sequential isolation of the most intense ions, up to five, depending on signal intensity, for fragmentation on the linear ion trap using collision induced dissociation at a target value of 100,000 charges. Target ions already selected for MS/MS were dynamically excluded for 60 seconds. General MS conditions were: electrospray voltage, 1.5 kV; no sheath and auxiliary gas flow. Ion selection threshold was 500 counts for MS/MS, and an activation Q-value of 0.25 and activation time of 30 ms were also applied for MS/MS.

# Data analysis

The raw files were imported into MaxQuant (Cox and Mann, 2008) software and default settings were used for the database search exception that the retention time alignment was performed. For the database search forward and reverse reviewed database from Uniprot (January, 2015 build) was used. The resultant summed intensities from MaxQuant for each protein were then then imported to Genedata Analyst v7.6 software (Genedata AG, Basel, Switzerland) and further statistical analysis was performed. Raw protein intensities for each sample were log transformed and median normalized. To filter for the significantly regulated proteins among the groups a paired Student T test was performed with the statistical cut-off value for fold change of 1.5 and *p* value of 0.05.

References:

Cox, J. and Mann, M. (2008) MaxQuant enables high peptide identification rates, individualized p.p.b.-range mass accuracies and proteome-wide protein quantification. *Nature Biotechnology* **26**: 1367–1372.

Pförtner, H., Wagner, J., Surmann, K., Hildebrandt, P., Ernst, S., Bernhardt, J., et al. (2013) A proteomics workflow for quantitative and time-resolved analysis of adaptation reactions of internalized bacteria. *Methods* **61**: 244–250.
