# Supplementary material for: Analyses of competent and non‐competent subpopulations of Bacillus subtilis reveal yhfW, yhxC and ncRNAs as novel players in competence
Source: Environ Microbiol. 2020 Apr 15;22(6):2312–28. doi: 10.1111/1462-2920.15005 (PMC7317962; doi:10.1111/1462-2920.15005)
Supplement: Supplementary file 5 — Appendix S5: Supporting information [file EMI-22-2312-s005.docx]

Samples for intracellular metabolome analysis were obtained by an extended fast vacuum dependent filtration. The filtration system should be turned on 15 min prior the sampling.

At every time point of interest, 20 OD units of culture was collected in a Falcon tube. For quenching of the metabolism, the cell culture was cooled down with liquid nitrogen directly after sampling (this step may only require seconds) but the sample should not be frozen. The sample was kept in and out of the nitrogen in alteration between 5-10 times. After transferring the cells to the filtration system (0.45 μm pore size; 100mm diameter Whatman® regenerated cellulose membrane filters RC55), the filter was washed with 2 times the volume of culture with 0.8% NaCl. The filter with cells was transferred into an extraction solution of cold ethanol 60% (w/v) with internal standards to a final concentration of 20 nmol (ribitol for gas chromatography mass spectrometry, GC-MS) and 2.5 nmol (camphorsulfonic acid for liquid chromatography mass spectrometry, LC-MS), shaken and immediately frozen in liquid nitrogen.

For cell disruption and metabolites extraction, a freeze/thaw cycle was performed by alternately thawing on ice, vortexing, and shaking the sample for 10 times to remove the cells from the filter. The sample was centrifuged for 5 min at 4 °C and 13000 rpm. The supernatant was transferred to a new 50 mL falcon and kept on ice. A second extraction was carried out by aqua dest. 5ml of water was added to the cell pellet The samples were shaken and mixed in alteration and centrifuged with the same parameters described previously. The aqueous extraction solution was combined with the ethanolic supernatant. The sample was filled with aqua dest. to an end concentration of ethanol of 10 % (filled to ~ 30 mL) and frozen at -80°C. The samples were lyophilized for analysis.

Ion pairing HPLC-MS was used to analyze intracellular nucleosides, nucleotides, sugar phosphates and co-factors. The measurement was conducted on a high-performance liquid chromatography (HPLC) system (1100 series, Agilent Technologies), coupled to a mass spectrometer (micrOTOF, Bruker Daltonics, Bremen, Germany). The lyophilized samples were dissolved in 100 uL of water HPLC grade and centrifuged for 2 min at room temperature. The supernatant was transferred into LC-vial for injection Metabolite quantification was done with the QuantAnalysis software package (Bruker Daltonics, Bremen, Germany). Intracellular amino acids and glycolytic intermediates were analyzed by GC-MS (GC 7890A, inert MSD 5979C, Agilent Technology). The dried samples were derivatized firstly with 60 µL of methoxyamine (20 mg/ml pyridine) for 90 min at 37⁰C and secondly with 120 µL of N-methyl-N-trimethylsilyltrifluroacetamide (Chromatographie-Service GmbH) for 30 min at 37⁰C. Samples were centrifuged for 2 min at room temperature and the supernatant was transferred into GC-vial for injection Metabolite quantification was conducted with the ChomaTOF software (LECO, St. Joseph, MI, USA).

The extracellular metabolome extraction was done with filtration of the supernatant at each time point of interest. 2 mL of culture was filtered through a 0.45μm pore size filter (Filtropur S, Sarstedt, Germany). The supernatant was frozen at -20°C until analysis.

1H Nuclear magnetic resonance (NMR) analysis was used to elucidate the extracellular metabolome. Supernatant culture sample was thawed at room temperature. A 400 µL sample was mixed with 200 µL of sodium hydrogen phosphate buffer (0.2 mM, pH 7.0) and trimethylsilyl propionic acid (TSP) (1 mM) made up with 50% D2O to provide a nuclear magnetic resonance 1H-NMR-lock signal.

The NMR spectra were obtained at 600.27 MHz at a temperature of 37°C (Bruker AVANCE-II 600, Bruker Biospin GmbH, Rheinstetten, Germany). A modified 1D-NOESY pulse sequence was used with presaturation on the residual HDO signal during both the relaxation delay and the mixing time. A total of 64 free induction decays (FID scans) were collected, using a spectral width of 30 ppm for a one dimensional spectrum.

Data analysis was done with AMIX v3.9.11 software (Bruker Biospin). The signal area of the internal standard trimethylsilyl propanoic acid was used for locking and the quant ref signal at 15ppm was used for quantification.
