# Supplementary material for: Analyses of competent and non‐competent subpopulations of Bacillus subtilis reveal yhfW, yhxC and ncRNAs as novel players in competence
Source: Environ Microbiol. 2020 Apr 15;22(6):2312–28. doi: 10.1111/1462-2920.15005 (PMC7317962; doi:10.1111/1462-2920.15005)
Supplement: Supplementary file 6 — Appendix S6: Supporting information [file EMI-22-2312-s006.docx]

| Strain | description | reference |
| --- | --- | --- |
| *B.subtilis 168* BFA1698 | Inactivation of *yhfW* by pMUTIN 4, strain created by Dr.Rob Meima | (Vagner et al., 1998), (Kobayashi et al., 2003) |
| *B.subtilis 168* BFA1701 | Inactivation of *yhxC* by pMUTIN 4, , strain created by Rob Meima | (Vagner et al 1998) (Kobayashi et al., 2003) |
| *B.subtilis 168* _P_*_comG-gfp_* | Chloramphenicol version created by Prof.Dr Jan-Willem Veening | (Smits et al., 2005) |
| *B.subtilis 168* *_Pspo0A-gfp_* |  | (de Jong et al 2010) |
| *B.subtilis 168* *_PsrfA-gfp_* | pSG1151 PsrfA-gfp constructed and transformed into B.subtilis 168 by Prof.Dr. Jan-Willem Veening |  |
| *B.subtilis 168* *_comK-gfp_* |  | (Smits et al., 2005) |
| *B.subtilis 168* _P_*_comG-gfp_- Δjag* | Deletion of *jag* using tetL from pBEST309 | This study |

de Jong,I.G., Veening,J.W., and Kuipers,O.P. (2010) Heterochronic phosphorelay gene expression as a source of heterogeneity in Bacillus subtilis spore formation. J Bacteriol 192: 2053-2067

Smits, W.K., Eschevins, C.C., Susanna, K.A., Bron, S., Kuipers, O.P., and Hamoen, L.W. (2005). Stripping Bacillus: ComK auto-stimulation is responsible for the bistable response in competence development. Mol. Microbiol. *56*, 604–614.

Vagner, V., Dervyn, E., and Ehrlich, S.D. (1998). A vector for systematic gene inactivation in Bacillus subtilis. Microbiol. Read. Engl. *144 ( Pt 11)*, 3097–3104.
